# Age-related molecular genetic changes of murine bone marrow mesenchymal stem cells

**DOI:** 10.1186/1471-2164-11-229

**Published:** 2010-04-07

**Authors:** Amber Wilson, Lina A Shehadeh, Hong Yu, Keith A Webster

**Affiliations:** 1 Department of Molecular and Cellular Pharmacology, and the Vascular Biology Institute, University of Miami School of Medicine, Miami, FL 33136, USA; 2 Veterans Administration Hospital, and the Vascular Biology Institute, University of Miami School of Medicine, Miami, FL 33136, USA

## Abstract

**Background:**

Mesenchymal stem cells (MSC) are pluripotent cells, present in the bone marrow and other tissues that can differentiate into cells of all germ layers and may be involved in tissue maintenance and repair in adult organisms. Because of their plasticity and accessibility these cells are also prime candidates for regenerative medicine. The contribution of stem cell aging to organismal aging is under debate and one theory is that reparative processes deteriorate as a consequence of stem cell aging and/or decrease in number. Age has been linked with changes in osteogenic and adipogenic potential of MSCs.

**Results:**

Here we report on changes in global gene expression of cultured MSCs isolated from the bone marrow of mice at ages 2, 8, and 26-months. Microarray analyses revealed significant changes in the expression of more than 8000 genes with stage-specific changes of multiple differentiation, cell cycle and growth factor genes. Key markers of adipogenesis including lipoprotein lipase, FABP4, and Itm2a displayed age-dependent declines. Expression of the master cell cycle regulators p53 and p21 and growth factors HGF and VEGF also declined significantly at 26 months. These changes were evident despite multiple cell divisions in vitro after bone marrow isolation.

**Conclusions:**

The results suggest that MSCs are subject to molecular genetic changes during aging that are conserved during passage in culture. These changes may affect the physiological functions and the potential of autologous MSCs for stem cell therapy.

## Background

Mesenchymal stem cells (MSCs) are pluripotent cells that have been reported to reside in virtually all postnatal organs and tissues (reviewed in [1-3]). They are defined by their ability to adhere to plastic, to differentiate into bone, cartilage and fat, and by expression of specific sets of cell-surface markers. The apparent plasticity of MSCs within the bone marrow and their similarity to subendothelial pericytes have lead to suggestions that these two cell types are closely related and possibly even the same [3]. Pericytes and actively proliferating MSCs both express alpha-smooth muscle actin (α-SMA), a marker of vascular smooth muscle cells, and both cell types reside within the domain of the microcirculation [3-7]. The pluripotential nature of MSCs has been demonstrated *in vitro *and *in vivo*. When systemically injected, mouse MSCs migrate to multiple tissues and differentiate into parenchymal cells of muscle, cartilage, skin, bone, liver, heart, brain, intestine and lung [8-19]. *In vitro*, defined conditions promote the differentiation of MSCs into skeletal muscle, endothelial cells, neurons, and cardiac myocytes in addition to bone, cartilage and fat [20-22]. It has been proposed that MSCs contribute to tissue and organ repair and have therapeutic potential in the regeneration or repair of multiple target tissues [23]. Several clinical trails have been launched to evaluate MSCs for the treatment of musculoskeletal, neurological and cardiovascular diseases [24,25].

The process of MSC aging is important from the perspective of tissue regeneration and repair because there is evidence that these beneficial functions may become handicapped with age. Age-related decline in the number of MSCs in the bone marrows of rodents, monkeys, and humans have been reported [26-33]. Most studies to date focused on the effects of aging on the ability of MSCs to enter osteogenic, chondrogenic and adipogenic programs. Some, but not all studies suggest that aging reduces osteogenesis and chondrogenesis while enhancing adipogenic potential [34-40]. These changes could provide an attractive explanation for the increased adiposity of bone marrow that is seen with age, and may be a factor in senile osteoporosis [41,42]. Other studies including some on humans suggest that the adipogenic potential of MSCs increases at mid-age but declines in old age [43]. Programs of senescence have been extensively studied particularly during passage of human MSCs, and these may provide clues to the mechanism of age-related decline of MSCs in the bone marrow [44]. However it is not known how aging affects growth factor, cell cycle or tumor suppressor genes despite the possible relevance to senescence and self-renewal. In fact to date there has been no comprehensive effort to analyze the effect of age on global gene expression of non-committed MSCs. In the present study, we harvested bone marrow from mice aged 2, 8, and 26 months, and obtained homogenous populations of MSCs from each age group. Comparisons of the transcription profiles of these MSCs reveal significant age-related changes in the expression of more than 8000 genes. We found that marker genes associated with adipogenic and osteogenic differentiation displayed a generalized decline with age. There were parallel declines of the cell cycle inhibitors p53 and p21, and the growth factors VEGF and HGF. These observations suggest that molecular genetic changes accumulate in bone marrow MSCs during aging that may affect functions, including differentiation and proliferation of these cells.

## Methods

### Cell culture and isolation

Mesenchymal stem cells (MSCs) were isolated from C57BL/6 WT mice aged 2, 8 and 26 months as described [45]. Briefly, femur and tibia were removed from both legs, four mice per age group, and the bone marrow flushed with culture medium using a syringe needle. The cells were filtered through a 70-micron strainer and centrifuged at 210 g for 10 minutes. Red Blood Cell Lysis Buffer (Sigma) was added, and the cells were plated on Falcon tissue culture plates in mouse mesenchymal stem cell basal media with supplements (Stem Cell Technologies, Va). Non-adherent cells were removed by rinsing and replacing the media after 48 hours and culture medium was replaced every 3 days. At 10 days post-harvest, the cells were removed with 0.25% Trypsin-EDTA solution (Gibco) and replated on new culture plates at a dilution of 1:2 (passage 1). Non-detached cells were discarded. Media was replaced twice weekly and cells were grown for 10 passages before harvest.

### Flow cytometry

Cells were resuspended at a density of 1.5 × 10^6 ^cells/mL in PBS containing 2% fetal bovine serum, 2 mM EDTA, and 0.1% sodium azide (FACS Buffer) and incubated at 4°C for 20 minutes with APC-or PE-conjugated antibodies against cell surface markers Sca-1, CD44, CD45, and CD11b (from Pharmingen, SanDiego, CA). Labeled cells were centrifuged, resuspended in 0.5 ml of FACS buffer, and analyzed using an LSRS1 flow cytometer and quantified with CELLQuest software.

### RNA isolation

Passage 11 MSCs from each age group were harvested at the same degree of confluence using identical procedures. RNA was purified using TriReagent (Sigma), and Qiagen RNeasy columns following the manufacturer's instructions. RNA integrity and concentration were analyzed by agarose-gel electrophoresis, UV NanoDrop spectrophotometry.

### Microarray

Equal amounts of RNA from 4 animals per age group were combined to generate a pooled sample for each experimental group. The pooled RNA samples were labeled for hybridization to the Affymetrix Mouse Genome 430 2.0 GeneChip Array, using standard Affymetrix protocols. This chip contains roughly 39,000 transcripts. A total of 11 chips were run including triplicates of 2-month samples and quadruplicates of 8 month and 12 month samples. Arrays were pre-hybridized with 1× Hybridization Buffer for 10 minutes at 45°C. The labeled samples were added to the GeneChip Arrays and hybridized for 16 hours at 45°C. The arrays were stained and washed according to Affymetrix Fluidics Station 450 protocol EukGEWS2v5_450. The intensity values were collected from the GeneChips by scanning the arrays with a GeneChip Scanner 3000 7G. The resultant images were analyzed with the MAS5 algorithm for quality control checks. Pearson correlation coefficients taken from plotting signal intensity values of duplicate chips across all genes validated that triplicate experiments were similar (typical correlation coefficients for previous double amplification experiments have been r2 ~.988). Chip intensity values were calculated using the gcrma algorithm. Chips were normalized with the quantile normalization procedure. Normalized expression values from the raw data were generated using default settings for the GC-Robust Multi-array Average (GC-RMA) method that provides the best balance of accuracy and precision [46] within GeneSpring (Silicon Genetics, Redwood City, CA). Subsequent statistical analysis was also performed in GeneSpring. The cross-gene error model was applied with replicates. The acceptance criterion for gene array expression changes was a minimum fold change of 2.0 and a t-test p-value of < 0.05. Venn diagrams and scatter plots were generated within GeneSpring.

### Western Blotting

Western blots were performed using previously described protocols [47]. Briefly, equal amounts of proteins were fractionated on 10% SDS-polyacrylamide gels and electroblotted to nitrocellulose (BioRad, Hercules, CA). Blots were stained with Ponceau Red to monitor the transfer of proteins. Membranes were blocked with 5% milk and incubated with p53 and actin antibodies (Santa Cruz Biotechnology, Santa Cruz, CA) overnight at 4°C. Blots were reacted with horseradish peroxidase-conjugated secondary antibodies and visualized by enhanced chemiluminescence (ECL, Pierce, Rockford, IL).

### Quantitative RT-PCR

RNA was reverse-transcribed using an RT2 PCR Array First Strand Kit (SuperArray, Frederick, MD) with random hexamers according to the manufacturer's instructions. Power SYBR Green PCR Master Mix (Applied Biosystems, Foster City, Ca) was used according to manufacturer's instructions. The reaction mixtures were run in 96-well RT2 ProfileTM PCR arrays (Mouse Cancer Pathway Finder and Mouse Osteogenic Pathway Finder, also from SuperArray) each containing primers for 90 genes. Detection was performed using an ABI Prism 7900HT FAST Sequence Detector System, and data analysis was carried out using the software provided. Cycle thresholds for each transcript (Ct) were related to the relative standard curve. Ct values were compared from different age groups and the mean and standard errors were calculated from two separate RNA extractions run in duplicate. Statistical analysis was carried out using a standard T-test.

### Differentiation assays

Osteogenesis: confluent monolayers of each cell group were incubated in osteoblast differentiation media containing DMEM-low glucose (Gibco), 10% heat-inactivated FBS, 1% penicillin-streptomycin, 10 mM beta-glycerophospate, 0.1 μM dexamethasone, and 0.2 mM ascorbic acid 2-phosphate [48]. The culture media was replaced every 3 days and after 14 days the cells were fixed with 4% paraformaldehyde and stained with Alizarin Red S (Sigma). Adipogenesis: confluent monolayers of each cell group were incubated in adipogenic induction medium consisting of DMEM-low glucose (Gibco) supplemented with 10% heat-inactivated fetal bovine serum (FBS), 1% penicillin-streptomycin, 1 μM dexamethasone, 0.5 mM IBMX, 100 μM indomethacin, and 10 Mg/mL insulin. After 6-days the medium was switched to adipogenesis maintenance media consisting of basal media 10% heat-inactive FBS, 1% penicillin-streptomycin, and insulin. Cultures were alternated weekly between induction media and maintenance media for 2 more weeks and then fixed in 4% paraformaldehyde and stained with Oil-Red-O (Sigma). Control plates were incubated in parallel with DMEM-low glucose supplemented with 10% heat inactivated FBS and 1% penicillin-streptomycin and subjected to the same fixing and staining procedures.

## Results

### Characterization of Bone Marrow Mesenchymal Stem Cells

**Figure 1:**
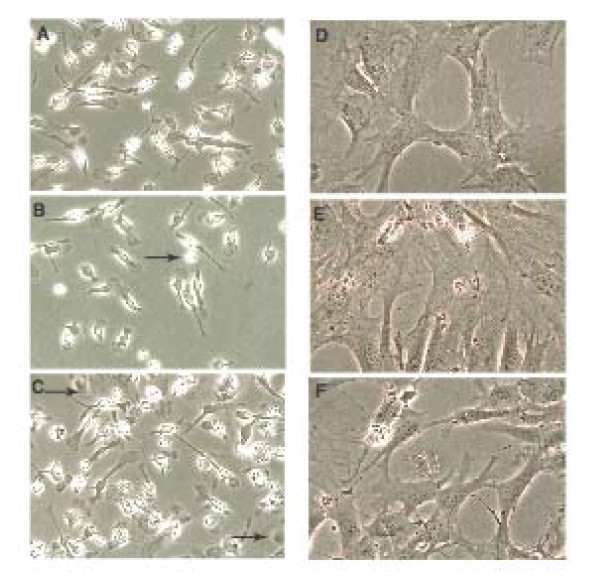
**Isolation of Bone Marrow Stem Cells. ** Bone marrow was aspirated from tibia and fibula of mice (4 per group) at age 2m, 8m, and 26m.  1(A-C) shows cells at the first passage from 2m, 8m, and 26m ages respectively.  Morphologies of the cells was heterogenous at this stage. 1(D-F) shows cells after 10 passages again a 2, 8 and 26m respectively, cells at this passage were homogeneous with a flattened morphology.

**Figure 2:**
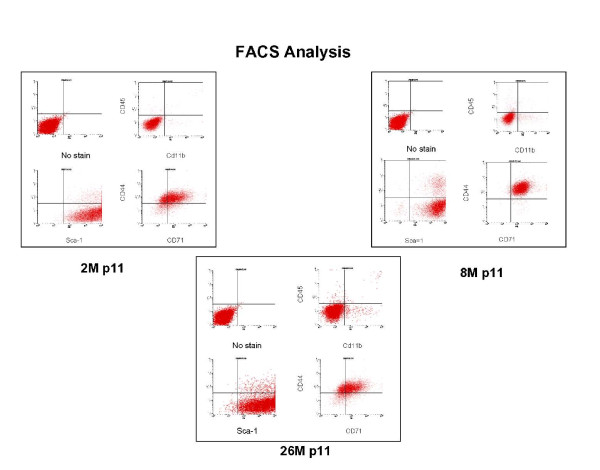
**FACS analyses indicate similar cell surface antigen profiles of MSC from different age groups.** Cells were labeled with fluorescent antibodies for Sca-1, CD44, CD11b, and CD45 and analyzed by FACS.  Consistent with a mesenchymal phenotype, all cells were positive for the markers Sca-1 and CD44 (>99% and 98% respectively).  Cells were negative for the monocyte marker CD11b and the hematopoietic lineage marker CD45 (<2%).

**Figure 3:**
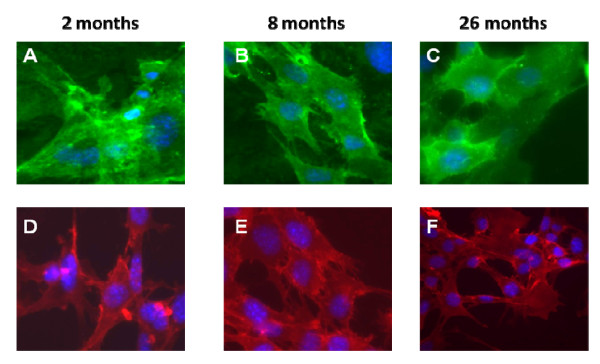
**Expression of stem cell markers.  **Immunofluoresence of isolated bone marrow stem cells.  Isolated MSC passage 11 were fixed and stained with anti-Sca-1 and CD44 antibodies.  All cells were positive for these stem cell markers.

In agreement with previous reports [44], early passage cultures were heterogeneous with cells displaying spindle-shaped, flat, and fibroblast-like morphologies (Figure 1(A-C)). During passage, cells with a flattened morphology were retained and were visually predominant by passage 11 (Figure 1(D-F). Passage 11 cells were characterized by FACS analysis for the expression of Sca-1, CD44, CD11b and CD45 (Figure 2). Sca-1 and CD44 are cell surface markers previously assigned to mouse MSCs. CD11b is a marker for granulocytes, monocytes, and natural killer cells and CD45 for hematopoietic lineage cells. Consistent with an MSC phenotype, all cells were Sca-1 and CD44 positive (>99% and ~80% respectively) and negative for CD11b and CD45 (both <2.0%). To confirm FACS cells were fixed stained with fluorescent lagged Sca-1 and CD44 antibodies. MSC from all ages were positive for these markers (Figure 3(A-F)).

### Age-related decline of MSC osteogenic differentiation

**Figure 4:**
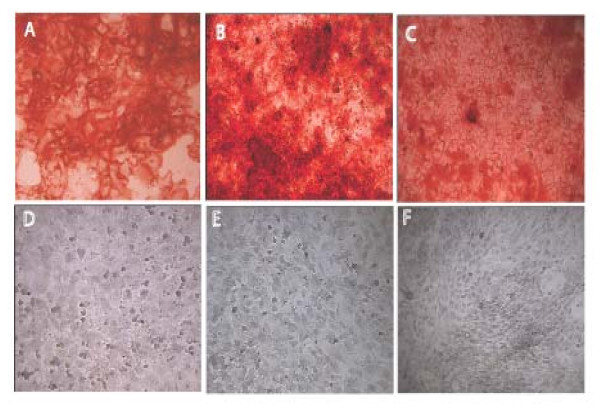
**Osteogenic Differentiation.** MSC, passage 11 from each age were grown to confluence and exposed to osteogenic induction medium for 14 days and stained with Alizarin Red. Cells from each age group were positive after 14 days (A-C; 2mo, 8mo, 26mo) whereas no staining was seen in cells treated with control medium (D,E, F; 2mo, 8mo, 26mo).

**Figure 5:**
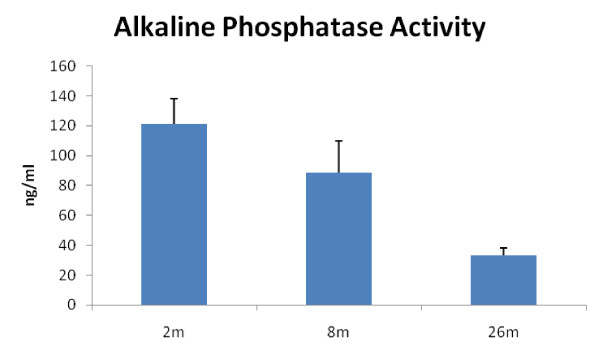
**Age-related Decline of Alkaline phosphatase (AP) activity.** Osteogenic differentiation involves increased secretion of AP.  Alkaline phosphataes was measured in the media of differentiating cell at day 10 as described in Methods.  Significantly less  AP was secreted from cells taken from mice at age 26 mo (n=4; p<0.01).

**Figure 6:**
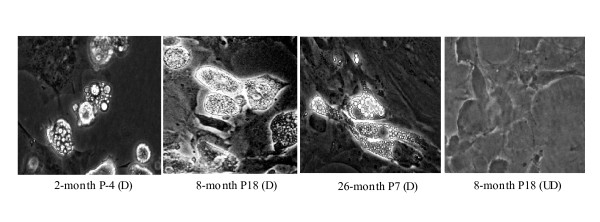
**Adipogenic differentiation. **MSCs from 2, 8, or 26-month old mice were grown to confluence and exposed to adipogenic induction medium (D) or culture medium (UD) as described in Methods.  Cell from each age group were Oli-Red positive and displayed intracellular fat droplettes.

Cultures from each age group were exposed to osteogenic differentiation as described in Methods. Figure 4 shows representative plates of cells stained with Alizarin Red S after differentiation treatments. All differentiated cultures stained positive (A-C) whereas no stain was detected in the controls (D-F). Microscopic visualization identified >95% of cells from each age as positive for Alizarin Red S after 14 days exposure to differentiation medium (data not shown). These results indicate that cells from all age groups were competent for osteogenic differentiation. To quantify osteogenic differentiation we measured secreted alkaline phosphates 10-days after exposure to differentiation medium as described in Methods. As shown in Figure 5 there was a progressive decline in alkaline phosphatase secreted, with 26-mo cells secreting ~25% compared with 2-mo cells. These results suggest that the potential for osteogenic differentiation declines with age. To determine whether the cells were competent for adipogenesis MSCs from each age group were exposed to adipogenic differentiation medium for 21 days, stained with Oil-Red-O and examined microscopically for cytoplasmic lipid droplets. Consistent with previous work in murine as well as human MSCs, we found that adipogenic differentiation decreased with passage number [48-51]. Clusters of cells with lipid droplets, positive for Oil-Red-O were found in MSCs of all age groups at passage number 7 or less (Figure 6). However adipogenic potential was lost with increasing passage; when MSCs from each age group were subjected to adipogenic differentiation conditions at passage > 14, only MSCs from 8-month old mice differentiated (see Figure 6). Interestingly MSCs from 8-month old mice also expressed the highest levels of adipogenic markers (see below).

### Distinct non-overlapping trends in gene expression during phases of aging

**Figure 7:**
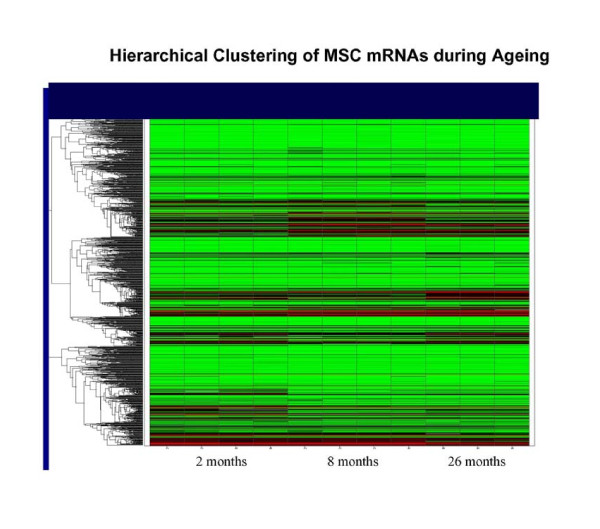
**Heirarchical Clustering of MSC mRNA.  **Clustering was implemented as described in Methods. Pearson correlation coefficients taken from plotting signal intensity values of duplicate chips across all genes validated that triplicate experiments were similar (typical correlation coefficients for previous double amplification experiments have been r2 ~ .988).

**Figure 8:**
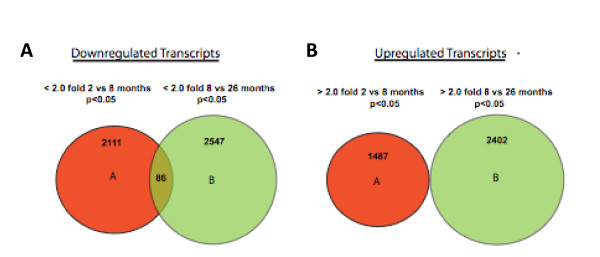
**Comparisons of down and up-regulated transcripts.  **Venn Diagram illustrating fold gene expression differences between 2m, 8m, and 26m (t-test <2-fold, p<0.01) of 40,359 transcripts. Expression levels of 2111 transcripts decreased from 2m to 8m and 2547 transcripts decreased from 8m to 26m.  Only 86 transcripts commonly decreased over both age groups. Analysis of up-regulated genes in the same manner revealed 1487 transcripts that were increased from 2m to 8m and 2402 increased from 8m to 26m.  There was no overlap.

**Figure 9:**
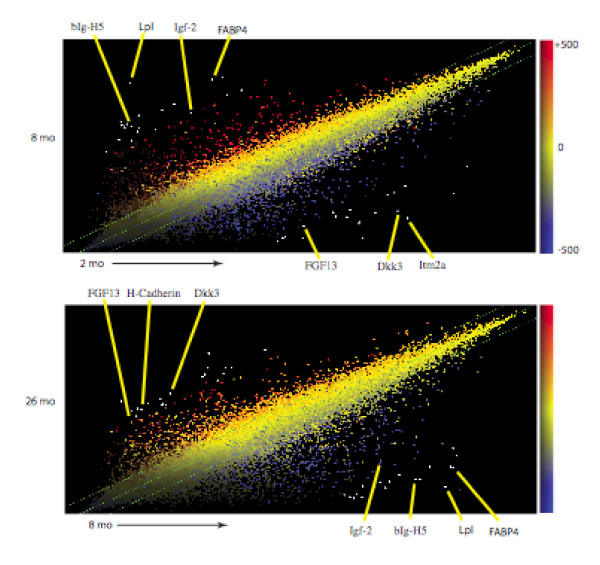
**Scatter Plot Analysis Indicating up and down regulated genes from microarray.  **This figure indicates the global changes in gene expression during two phases of aging and growth.  While the two figures look similar, the identification of the genes reveals two different gene sets.  Some of the genes that undergo some of the largest changes are indicated.

Results of microarray analyses using the Affymetrix Mouse Genome 430 2.0 are shown in Figures 3 and 4 and Tables 1, 2, and 3 (see Additional files 1, 2, 3). Hierarchical clustering of mRNAs from each set of time points indicated excellent reproducibility between samples (Figure 7). Venn diagrams of differentially expressed genes using a threshold of 2-fold are shown in Figure 8. 2111 transcripts declined between 2 and 8 months and 2547 transcripts between 8 and 26 months. Eighty-six transcripts corresponding to 71 genes were common to both groups. 1487 transcripts were elevated in the 2- to 8-month group and 2402 in the 8- to 26-month group; there was no overlap between these groups. The low degree of overlap between groups indicates that transcripts that change significantly during 2-8 months do not continue to change in the same direction during 8-26 months, but rather remain at the 8-month level, whereas different sets of transcripts change during 8-26 months. Scatter plots indicating up and down-regulated genes are shown in Figure 9.

### Age-related trends in differentiation, growth factor and cell cycle genes by cluster analysis

#### Differentially expressed genes between 2 and 8 months

A series of osteogenic, homeobox, and integrin gene transcripts decreased significantly between 2 and 8-months, see Table 1 (Additional File 1). The osteogenic markers included, osteoadherin (20-fold), periostin osteoblast specific factor (20-fold), osteoglycin (7-fold), osteonectin (4-fold), osteonitogen (4-fold), and osteoblast stimulating factor (2-fold). Down-regulated integrins included, integrin α-4 (62-fold by RT-PCR), α-6 (5-fold), α-8 (10-fold), α-10 (5-fold), and β-1 (3-fold by RT-PCR); integrin linked kinase (ilk) and Adams-5 also decreased by 3-fold. Adams are transmembrane proteins that have metalloprotease, integrin-binding, intracellular signaling and cell adhesion activities [52]. Down-regulated homeobox genes included homeobox msh-like (50-fold), homeobox B2 (30-fold), paired-related homeobox (30-fold), homeobox B6 (25-fold), homeobox 5 (10-fold), and HoxB9 (15-fold). The levels of TGFβ signaling pathway transcripts TGF-β2 and the receptor endoglin increased. Endoglin is an accessory receptor for several growth factors of the TGFβ family that is expressed in adult bone marrow hematopoietic stem cells [53,54]. IGF-1BP3 and IGF-2BP4 transcripts were also increased between 2 and 8 months. There were increases of lipoprotein lipase (Lpl) and collagen type-VIIa (Col7a). Lpl is a marker of adipogenesis; Col7a is a component of the epidermal basement membrane [55].

#### Differentially expressed genes between 8 and 26 months

Table 2 (Additional File 2) shows the top 80 down-regulated transcripts in the aging (8-26 mo) group. These include 3 glutathione-S-transferase genes and glutathione peroxidase, suggesting possible down-regulation of this anti-oxidant pathway. There were also 3 separate hits for serine (or cysteine) proteinase inhibitor (clades A, B and E), calcium-activated chloride channels, and solute carrier family-38 genes. The osteogenic and homeobox markers, that declined between 2-8 months remained depressed at 26 and the levels of two key adipogenesis marker genes Lpl and FABP4 decreased markedly between 8 and 26-months. Gene transcripts that were significantly increased between 8 and 26 months included H-cadherin, Col7a, and several bone morphogenic protein (BMP) gene family transcripts including BMP2 (3.8-fold), BMP3 (2.5-fold), BMP4 (6.5-fold), Bmp receptor 1b (3-fold), BMP2 inducible kinase (3-fold), BMP activation membrane bound inhibitor (Bambi, 20-fold), and BMP-binding endothelial receptor (Bmper, 10-fold).

#### Cell cycle regulators and apoptosis

**Figure 10:**
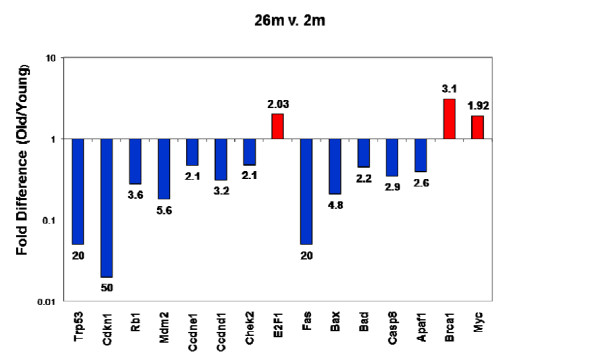
**RT-PCR confirmation of age-related changes of cell cycle and apoptosis genes.  **A RT-PCR (cancer) SuperArray of 84 cell cycle and apoptosis genes was used to compare mRNA from 2m and 26m mouse MSCs.  The analysis confirms 2-fold and 50-fold decrease of p53 and p21 respectively and significant decrease of apoptosis transcripts with age.

**Figure 11:**
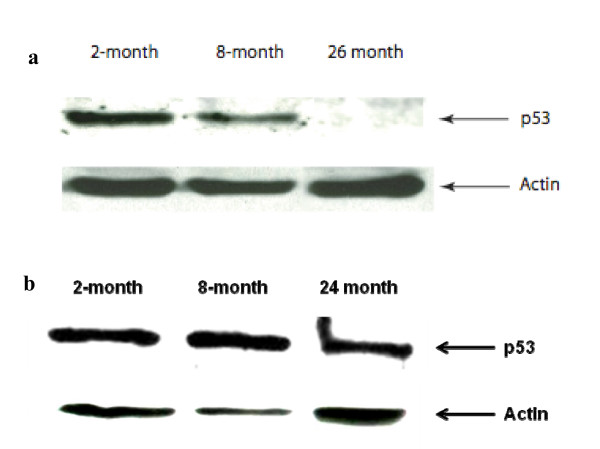
**Western blot of p53 expression.  **(a) Cell lysates from each age group were analyzed by western blot as described in Methods.  Consistent with microarray and RT-PCR analysis, MSCs derived from 26-month mice did not express the p53 protein.  (B) Spleen lysates from progressively aged mice showed no change of p53 protein.

**Figure 12:**
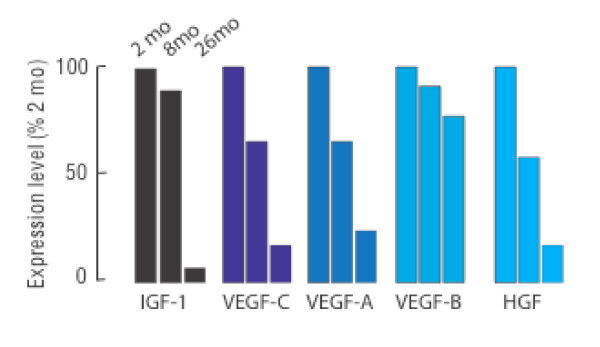
**Microarray profiles of pro-angiogenic cytokines and growth factor genes.  **Expression levels of IGF-1, VEGF-A, VEGF-B, VEGF-C and HGF were from microarray.

Microarray analysis revealed significant declines in the transcripts encoding cell cycle regulators Trp53 (p53), Cdkn1a (p21), CHEK2 and retinoblastoma gene product (Rb1), and multiple apoptosis genes over the entire 2-26 month period. These trends were confirmed by rtPCR using a cancer pathway SuperArray, (Figure 10 and Table 3 (Additional file 3)). P53 and p21 transcript levels decreased by 26 and 50-fold respectively. CHEK2 transcripts decreased by 2-fold and Rb1 by 4-fold. Fas decreased by 2-fold and Bax, Bad, Caspase 8, and Apaf1 by 2-4-fold. When the same SuperArray was used to compare transcripts from hearts of 2-month versus 24-month mice only minor age-related changes were observed in these transcript (data not shown). Western analyses were implemented to determine whether the changes in p53 were reflected at the protein level. As shown in Figure 11, p53 was reduced at 8-months compared with 2-months and was almost undetectable at 26 months. In contrast there was no change of p53 expression in the spleen of 2 month versus 24-month mice (Figure 12).

#### Growth factors

**Figure 13:**
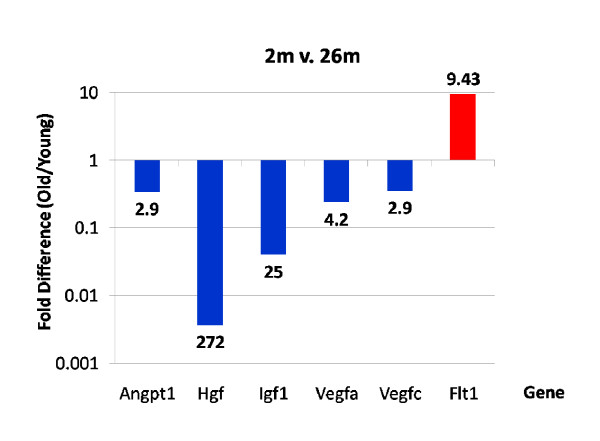
**RT-PCR measurement of Growth factor transcripts.   **RT-PCR was performed as described in Methods.  These analysis confirmed marked down regulation of the growth factor cytokine genes with age.  The largest changes were the HGF gene (>272-fold) and IGF-1 gene transcripts.

**Figure 14:**
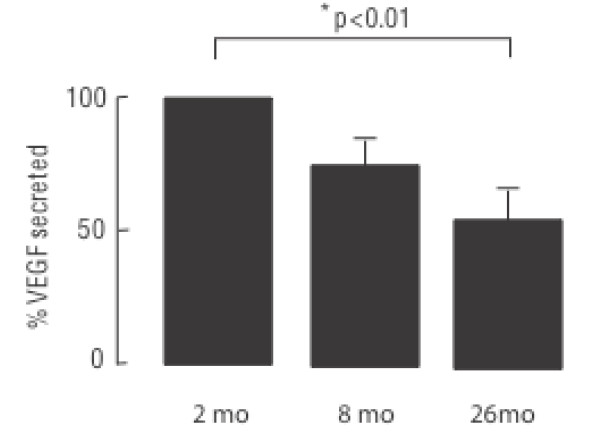
**ELISA measurement of VEGF secretion.   **ELISA was performed as described in Methods.  MSC from each age were grown to confluence in parallel and VEGF measured in the medium 24h after replacement. Significantly less VEGF was secreted from cells taken from mice at age 26 mo (n=4; p<0.02).

Microarray analysis identified five pro-angiogenic growth factor genes with decreased expression during aging, HGF, IGF-1, VEGF-A and C and angiopoietin-1 (ang-1). These changes were confirmed by rtPCR (Figure 13 and 14). E2F1 and the VEGF receptor Flt1 were elevated in 26-month cells (E2F1, 2-fold; Flt1, 9-fold by RT-PCR). It is noteworthy that all of the gene expression changes detected by the microarray analysis that were represented in the RT-PCR arrays *w*ere confirmed, and quantification of individual transcripts was usually within the same range. To confirm mRNA transcript results we measured VEGF protein levels by ELISA (Figure 14). VEGF secretion by 26-month MSCs was significantly reduced in 8-and 26-month cells.

## Discussion

We compared global gene transcriptional profiles of uncommitted bone marrow derived mesenchymal stem cells from mice at 3 different ages and across 2 intervals of the murine lifespan. Changes of gene expression occurring between 2 and 8 months have little in common with those between 8 and 26 months. The small overlap of down-regulated transcripts (87/2111) and no overlap of up-regulated genes (0/2547), is consistent with the biologically distinct stages represented by these time periods that correspond roughly to young, mature, and aged. An important and novel aspect of this study is that distinctive patterns of gene expression were apparent between the cells despite 11 passages and culture for 6-weeks in defined MSC proliferation medium. The cells were cultured in parallel under identical conditions, harvested at the same degree of confluence and displayed homogeneity as reflected by similar cell-surface markers at the time of RNA isolation. Therefore, it seems likely that the differences in transcript profiles are age-related and reflect molecular genetic modifications that are retained during cell division.

Despite reciprocal changes in the transcript levels of multiple genes including those associated with TGF-β and IGF-1 signaling pathways over the 2-8 month and 8-26 month periods, there was an overall trend of decreased osteogenic and adipogenic marker expression over the entire 2- to 26-month period of aging. The increase of Lpl transcripts apparent between 2 and 8 months was lost at 26 months in parallel with a dramatic decline of FABP4 transcripts. The osteogenic and homeobox markers that declined between 2 and 8 months remained depressed at 26 month. Several bone morphogenic protein (BMP) family transcripts including BMP-2, -3 and -4, Bmp receptor-1b, and BMP activation membrane bound inhibitor were elevated at 26 months. However multiple other osteogenic markers that declined in the 2-8 month age group did not recover. Integrins and smooth muscle-related transcripts (α-SMC and γ-SMC) decreased during 2-8 months and increased again at 26 months, whereas transcripts of Sfrp1, a key component of Wnt signaling, and a possible modulator of osteogenic versus adipogenic differentiation [56] decreased progressively. These results are consistent with declines in osteogenic and perhaps adipogenic potential with age, although passage number may be more important than age for the latter. The results are also consistent with previous reports that osteogenesis and adipogenesis decline with age and passage of murine MSCs [48-51]. The situation may be different in humans where MSCs from aged subjects display more rapid senescence in culture, but do not appear to have reduced osteogenic or adipogenic differentiation potentials at least at early passage in culture [57-59].

As noted above, only 86 gene transcripts were down regulated over the entire 2-26 month period. Importantly, these down-regulated transcripts included multiple cell cycle and growth factor genes. VEGF, HGF and IGF-1 were all significantly decreased in MSCs from 26-month old mice relative to cells from either 2 or 8-months (Figure 12). RT-PCR confirmed 4- and 3-fold decreases of VEGF-A and -C respectively, 272-fold decrease of HGF, and 22-fold decrease of IGF-1 transcripts over 2-26 months (Figure 13). ELISA further confirmed the decrease of VEGF secretion of cells from aged mice (Figure 14. The extensive decrease of HGF transcripts suggests that basal expression of the HGF gene is largely extinguished in cells from aged mice. Such changes may adversely affect both the survival and angiogenic potential of MSCs from aged bone marrow. This in turn might negatively influence the repair functions as well as the therapeutic potential of these cells, particular as they relate to wound healing and treatment of cardiovascular disease where neo-angiogenesis is essential.

The decreased expression of the p53/p21 cell cycle checkpoint pathway in 26-month MSCs was confirmed by RT-PCR and western blot. P53 is normally stabilized when cells are exposed to conditions that promote DNA damage when it translocates to the nucleus and activates the transcription of p21 (cyclin-dependent kinase inhibitor-1a) [60]. P21 inhibits the cyclin-dependent kinase 2 (CDK2) causing cell cycle arrest in G1. If DNA is successfully repaired, p53 is degraded and cell division can be restarted. In somatic cells the p53/p21 pathway is progressively activated during aging as telomeres are lost and cells accumulate DNA damage, eventually promoting senescence [61]. Conversely, the p53-pathway is inactive in >50% of oncogenically transformed cells accounting at least in part for the resistance of these cell to senescence [62]. In embryonic stem cells (ESC) p53 is expressed at low levels and does not induce p21 because translocation to the nucleus is blocked (reviewed in 63). Therefore ESCs also evade p53-mediated senescence. Genome integrity in ESCs is maintained by enhanced telomerase activity, efficient DNA repair, and highly active p53-independent apoptosis [64]. Our observations that aged MSC lost expression of p53 and p21, as well CHEK2, may explain how these cells avoid age-related senescence. Loss of Rb1 may also contribute to this; Rb, p21 and p53 are all required for replicative senescence of primary somatic cells [65,66]. These results may also be quite relevant to oncogenesis; an age-related loss of p53 may predispose these cells to oncogenic transformation, perhaps generating cancer stem cells [67]. Aging is a well-established risk factor for oncogenesis [68,69]. The relevance of Flt1 induction is not clear, however it has been reported that bone marrow derived hematopoietic progenitor cells that express high levels of Flt1 have an enhanced potential to home to tissues that express VEGF [70].

There have been conflicting reports on the effect of age on the adipogenic potential of MSC (reviewed in [1-3]). Muraglia et al [35] isolated 185 clones of human MSCs and showed that 184 of these differentiated along an osteogenic lineage, whereas fewer clones showed chondrogenic or adipogenic potential, and the potentials of the latter decreased with passage number. MSC from human adipose were also reported to become more osteogenic at late passage [38]. Other studies suggest that age affects senescence but not differentiation potential of human MSCs [57-59]. MSC from rats rapidly loose chondrogenic potential during aging from immature to mature or old, and this is paralleled by lower basal expression of related genes [37]. There is also evidence that mesenchymal stem cells committed to an adipogenic program can be induced to trans-differentiate to the osteogenic pathway and vice versa [71]. Moreman et al [36] reported recently that freshly isolated bone marrow of 26-month old mice contains significantly greater numbers of cells committed to the adipogenic lineage than does the bone marrow of 8-month mice. These changes were paralleled by increased adipogenic and decreased osteogenic marker gene transcripts. These studies do not necessarily conflict with our findings because Moreman et al studied freshly isolated cells whereas we used passaged cells. Culture in vitro may eliminate or reverse the programming of cells that are committed at the time of isolation [71]. We found that transcript levels of osteogenic markers decreased between 8 and 26 months whereas adipogenic markers increased at 8-months but decreased at 26-months. Differentiation studies were consistent with a biological consequence of these changes (Figure 2). These trends are reminiscent of the aging process in humans where adiposity tends to increase at mid-age while both adiposity and osteogenicity decrease during old age [72,73]. Of note mice aged 8-months are equivalent to a human age between 30-40 whereas a 26-month old mouse is equivalent to >70 human years [74].

## Conclusions

Our studies indicate dramatic changes in the expression of multiple genes during aging with some of the greatest fluctuations represented by adipogenic and osteogenic markers, growth factors and cell cycle regulator genes. Major trends included higher adipogenic gene markers and lower osteogenic markers at 8-months compared with 2-months, loss of adipogenic markers at 26-months, and globally decreased transcripts for growth factors and cell cycle regulators p53 and p21 over the entire aging period. The results suggest that maturation and aging of the bone marrow define distinctive gene expression patterns that effect both tissue-specific and housekeeping genes. The loss of growth factor, survival, and cell cycle control genes implies that aged MSCs may loose some of their migration and repair properties while avoiding age-induced senescence. The retention of age-determined expression profiles of tissue specific genes during passage *in vitro *suggests that certain gene sets may be irreversibly affected by aging *in vivo*, such that they are refractive to reprogramming signals after isolation.

## Authors' contributions

AW implemented cell culture, RNA isolation and characterization of phenotypes. LAS implemented bioinformatics analyses. HY participated in the design of the study. KAW conceived of the study, and directed implementation, analyses and presentation. All authors read and approved the final manuscript.

## Supplementary Material

Additional file 1 **Table 1**. Fold change of individual transcript levels are from microarray. Click here for file

Additional file 2 **Table 2**. Eighty most down-regulated transcripts 8-26 months. Click here for file

Additional file 3 **Table 3**. Changes of cell cycle and growth factor gene transcripts over 2-26 months. Click here for file
