# Supplementary material for: Age-related molecular genetic changes of murine bone marrow mesenchymal stem cells
Source: BMC Genomics. 2010 Apr 7;11:229. doi: 10.1186/1471-2164-11-229 (PMC2873471; doi:10.1186/1471-2164-11-229)
Supplement: Additional file 1 — Table 1. Fold change of individual transcript levels are from microarray. [file 1471-2164-11-229-S1.DOC]

Table 1 Age-related changes of clustered gene transcripts.

Fold change of individual transcript levels are from microarray (n refers to the number of hybridizing sequences used to calculate fold change; * indicates transcripts that are confirmed by RT-PCR. For gene groups (osteogenic markers, homeobox, integrins, BMP) the number in brackets refers to the number of individual related transcripts represented in each group.

Table 1a

**2-8mo up**

Fold Change

*TGF-β2 3 (n=2; p<0.01)

*TGFβR3 (endoglin) 5.3 (n=2; p<0.01)

*Col7a 52 (p<0.001)

Sfrp1 30 (n=5; p<0.001)

*IGF2-BP3 30 (n=6; p<0.001)

*IGF-BP4 7 (n=6; p<0.001)

Lpl >100 (n=4; p<001)

**2-8mo down**

*Osteogenic markers (7) 5-40

Homeobox (11) 5-50

*Integrins (7) 4-10

Table 1b

**8-26mo up**

H-cadherin 43 (n=2;p<0.001)

*Integrins (7) 3-30

*BMP (7) 4-20

*SMAD4 2.2 (p<0.001)

**8-26mo down**

*FABP4 >100 (n=3;p<0.001)

*TGFβ2 2 (n=2;p<0.01)

*TGFβR3 (endoglin) 8 (n=2;p<0.01)

*Col7a 45 (p<0.001)

Sfrp1 65 (n=5;p<0.001)

*IGF2-BP3 30 (n=6;p<0.001)

*IGF1-BP4 25 (n=6;p<0.001)

Lpl >100 (n=6;p<0.001)
