# Supplementary material for: Age-related molecular genetic changes of murine bone marrow mesenchymal stem cells
Source: BMC Genomics. 2010 Apr 7;11:229. doi: 10.1186/1471-2164-11-229 (PMC2873471; doi:10.1186/1471-2164-11-229)
Supplement: Additional file 2 — Table 2. Eighty most down-regulated transcripts 8-26 months. [file 1471-2164-11-229-S2.DOC]

Table 2. Eighty most down-regulated transcripts 8-26 months.

| Fold Decrease | Gene Name | Common | Description |
| --- | --- | --- | --- |
| 1234.356 | 1415904_at | Lpl | lipoprotein lipase |
| 634.4519 | 1417023_a_at | Fabp4 | fatty acid binding protein 4, adipocyte |
| 497.0000 | 1415897_a_at | Mgst1 | microsomal glutathione S-transferase 1 |
| 488.6598 | 1427183_at | Efemp1 | EGF-containing fibulin-like extracellular matrix protein 1 |
| 318.9616 | 1419728_at | Cxcl5 | chemokine (C-X-C motif) ligand 5 |
| 271.8531 | 1426454_at | Arhgdib | Rho, GDP dissociation inhibitor (GDI) beta |
| 268.3215 | 1448881_at | Hp | Haptoglobin |
| 225.2782 | 1460259_s_at | Clca1 | chloride channel calcium activated 2 |
| 220.1863 | 1451335_at | Plac8 | placenta-specific 8 |
| 219.1601 | 1428111_at | Slc38a4 | solute carrier family 38, member 4 |
| 218.0465 | 1416666_at | Serpine2 | serine (or cysteine) proteinase inhibitor, clade E, member 2 |
| 215.1656 | 1454268_a_at | Cyba | cytochrome b-245, alpha polypeptide |
| 163.2813 | 1424923_at | Serpina3g | serine (or cysteine) proteinase inhibitor, clade A, member 3G |
| 153.5844 | 1460187_at | Sfrp1 | secreted frizzled-related sequence protein 1 |
| 145.1407 | 1417852_x_at | Clca1 | chloride channel calcium activated 1 |
| 133.9854 | 1451791_at | Tfpi | tissue factor pathway inhibitor |
| 130.6383 | 1427760_s_at | Mrpplf4 | Proliferin |
| 126.4167 | 1430388_a_at | Sulf2 | sulfatase 2 |
| 109.0524 | 1449106_at | Gpx3 | glutathione peroxidase 3 |
| 87.33766 | 1422671_s_at | Naalad2 | N-acetylated alpha-linked acidic dipeptidase 2 |
| 85.01071 | 1418990_at | Ms4a4d | membrane-spanning 4-domains, subfamily A, member 4D |
| 81.90377 | 1422317_a_at | Il1rl1 | interleukin 1 receptor-like 1 |
| 81.33333 | 1453152_at | Mamdc2 | MAM domain containing 2 |
| 79.77174 | 1448889_at | Slc38a4 | solute carrier family 38, member 4 |
| 77.84188 | 1423135_at | Thy1 | thymus cell antigen 1, theta |
| 77.47440 | 1416846_a_at | Pdzrn3 | semaF cytoplasmic domain associated protein 30 |
| 76.75197 | 1449630_s_at | Mark1 | MAP/microtubule affinity-regulating kinase 1 |
| 74.97696 | 1419456_at | Dcxr | dicarbonyl L-xylulose reductase |
| 69.03030 | 1440085_at | Xedar | ectodysplasin A2 isoform receptor |
| 68.99351 | 1434913_at | Hmgcll1 | 3-hydroxymethyl-3-methylglutaryl-Coenzyme A lyase-like 1 |
| 68.17114 | 1418422_at | Serpinb9f | serine (or cysteine) proteinase inhibitor, clade B, member 9f |
| 66.27208 | 1448152_at | Igf2 | insulin-like growth factor 2 |
| 65.26185 | 1448499_a_at | Ephx2 | epoxide hydrolase 2, cytoplasmic |
| 55.55024 | 1434470_at | Syt13 | synaptotagmin 13 |
| 55.34783 | 1417256_at | Mmp13 | matrix metalloproteinase 13 |
| 55.26570 | 1417732_at | Anxa8 | annexin A8 |
| 54.66425 | 1448123_s_at | Tgfbi | transforming growth factor, beta induced |
| 54.30052 | 1448754_at | Rbp1 | retinol binding protein 1, cellular |
| 51.24161 | 1452291_at | Centd1 | centaurin, delta 1 |
| 51.18483 | 1426758_s_at | Gtl2 | GTL2, imprinted maternally expressed untranslated mRNA |
| 49.97821 | 1420349_at | Ptgfr | prostaglandin F receptor |
| 49.33202 | 1421228_at | Ccl7 | chemokine (C-C motif) ligand 7 |
| 48.72973 | 1420380_at | Ccl2 | chemokine (C-C motif) ligand 2 |
| 46.11511 | 1450663_at | Thbs2 | thrombospondin 2 |
| 46.09865 | 1415856_at | Emb | Embigin |
| 45.13043 | 1418061_at | Ltbp2 | latent transforming growth factor beta binding protein 2 |
| 42.75923 | 1453924_a_at | Ptgfr | prostaglandin F receptor |
| 42.39051 | 1421074_at | Cyp7b1 | cytochrome P450, family 7, subfamily b, polypeptide 1 |
| 41.26316 | 1437689_x_at | Clu | Clusterin |
| 40.73073 | 1424775_at | Oas1g | 2'-5' oligoadenylate synthetase 1G |
| 40.70815 | 1424433_at | Mrsb | methionine sulfoxide reductase B |
| 39.95683 | 1417466_at | Rgs5 | Regulator of G-protein signaling 5. |
| 39.9177 | 1435603_at | SST3 | secreted protein SST3 |
| 39.56216 | 1455393_at | Cp | Ceruloplasmin |
| 39.32353 | 1422571_at | Thbs2 | thrombospondin 2 |
| 38.99153 | 1425145_at | Il1rl1 | interleukin 1 receptor-like 1 |
| 38.96104 | 1452968_at | Cthrc1 | Collagen triple helix repeat containing protein. |
| 38.92208 | 1425357_a_at | Grem1 | cysteine knot superfamily 1, BMP antagonist 1 |
| 38.79508 | 1418186_at | Gstt1 | glutathione S-transferase, theta 1 |
| 37.27273 | 1433992_at | Apxl | apical protein, Xenopus laevis-like |
| 36.97509 | 1416286_at | Rgs4 | regulator of G-protein signaling 4 |
| 36.49842 | 1438404_at | Rnf144 | ring finger protein 144 |
| 35.81431 | 1421063_s_at | Snurf | small nuclear ribonucleoprotein N |
| 34.93282 | 1449434_at | Car3 | carbonic anhydrase 3 |
| 34.70032 | 1422788_at | Slc43a3 | solute carrier family 43, member 3 |
| 34.43540 | 1425510_at | Mark1 | MAP/microtubule affinity-regulating kinase 1 |
| 34.18738 | 1422789_at | Aldh1a2 | aldehyde dehydrogenase family 1, subfamily A2 |
| 34.00468 | 1419043_a_at | AW111922 | expressed sequence AW111922 |
| 34.00428 | 1454764_s_at | Slc38a1 | solute carrier family 38, member 1 |
| 33.50291 | 1433919_at | Asb4 | ankyrin repeat and SOCS box-containing protein 4 |
| 33.46749 | 1436999_at | AL024069 | expressed sequence AL024069 |
| 33.19337 | 1435943_at | Dpep1 | dipeptidase 1 (renal) |
| 31.95652 | 1436870_s_at | AU041783 | expressed sequence AU041783 |
| 31.29187 | 1423756_s_at | Igfbp4 | insulin-like growth factor binding protein 4 |
| 30.98855 | 1423891_at | Gstt3 | glutathione S-transferase, theta 3 |
| 30.97368 | 1415857_at | Emb | Embigin |
| 30.89928 | 1455223_at | Igf2bp1 | IGF2 binding protein 1 |
| 30.05910 | 1419834_x_at | Mark1 | MAP/microtubule affinity-regulating kinase 1 |
| 29.65289 | 1417853_at | Clca2 | chloride channel calcium activated 1 |
| 29.18410 | 1418949_at | Gdf15 | growth differentiation factor 15 |
