# Supplementary material for: Age-related molecular genetic changes of murine bone marrow mesenchymal stem cells
Source: BMC Genomics. 2010 Apr 7;11:229. doi: 10.1186/1471-2164-11-229 (PMC2873471; doi:10.1186/1471-2164-11-229)
Supplement: Additional file 3 — Table 3. Changes of cell cycle and growth factor gene transcripts over 2-26 months. [file 1471-2164-11-229-S3.DOC]

Table 3. Changes of cell cycle and growth factor gene transcripts over 2- 26 months.

RT-PCR assays were implemented as described in Methods. Numbers indicate fold changes of RNA isolated from MSCs at passage 12.

Table 3

**2-26mo down**

Fold Change

p53 26 (p<0.001)

p21 50 (p<0.001)

Chek2 2 (p<0.001)

Rb1 4 (p<0.001)

IGF-1 22 (p<0.001)

HGF >100 (p<0.001)

VEGFA 4 (p<0.001)

VEGFC 3 (p<0.001)

**2-26mo up**

E2F1 2 (p=0.004)

Flt1 9 (p<0.001)
